# Early versus delayed trial without catheter in men with Acute urinary retention - Study Protocol for a dutch national randomized trial

**DOI:** 10.1371/journal.pone.0354879

**Published:** 2026-08-06

**Authors:** Liselot L.A. Ribbert, Nadine A.M. van Merode, Marco H. Blanker, Michelle M.A. Kip, Ingrid M. Nijholt, Lambertus P.W. Witte, on behalf of the RELIEF Study Group

**Affiliations:** 1 Department of Primary and Long-term Care, Cure and Care in the Community Context (FOUR-C)-research Program, University Medical Center Groningen (UMCG), Groningen, The Netherlands; 2 Department of Urology, Isala Clinics, Zwolle, The Netherlands; 3 Department of Urology, Maastricht University Medical Center+, Maastricht, The Netherland; 4 Department of Health Technology & Services Research, Faculty of Behavioral Management and Social Sciences, Technical Medical Centre, University of Twente, Enschede, The Netherlands; Jan Biziel University Hospital No 2 in Bydgoszcz: Szpital Uniwersytecki Nr 2 im dr Jana Biziela w Bydgoszczy, POLAND

## Abstract

**Introduction:**

Acute urinary retention (AUR) is a burdensome urological emergency, often caused by benign prostatic obstruction. Standard management includes transurethral catheterization, alpha-blocker therapy, and a trial without catheter (TWOC) to assess spontaneous voiding. Despite the high incidence of AUR, the optimal catheter duration prior to TWOC remains uncertain. Evidence is limited, guideline recommendations are largely expert-based, and catheter duration varies widely in practice. Prolonged catheterization may increase morbidity and discomfort, whereas earlier removal may raise concerns regarding recurrent retention.

**Methods:**

RELIEF is a pragmatic, multicenter, randomized non-inferiority trial comparing TWOC on day 3 versus TWOC on day 14 in men with AUR. All participants undergo transurethral catheterization and receive alpha-blocker therapy prior to randomization. Primary outcome is TWOC success, defined as a voided volume ≥100 mL and post-void residual ≤200 mL. The primary analysis follows a per-protocol approach, with a complementary intention-to-treat analysis to confirm non-inferiority. Secondary outcomes include recurrent retention, catheter-related complications, patient-reported symptoms and quality of life, and cost-effectiveness from healthcare and societal perspectives over 18 months. In parallel, a qualitative substudy using semi-structured interviews with healthcare professionals aims to explore the determinants underlying current practice variation, as well as the perceived barriers and facilitators for the sustainable implementation of a standardized TWOC strategy in future clinical practice.

**Discussion:**

By comparing two catheterization intervals already applied in routine care and combining clinical, patient-reported, economic, and implementation outcomes, RELIEF is designed to generate directly applicable evidence on TWOC timing after AUR. The results are expected to support guideline refinement and improve consistency of care.

**Trial registration:**

WHO ICTRP primary registry, identifier NL-OMON57836. Registered on February 7, 2025.

## 1. Introduction and rationale

Acute urinary retention (AUR) is a common and painful urological emergency, most often caused by bladder outlet obstruction (BOO) secondary to benign prostatic hyperplasia (BPH) [1,2]. BOO is highly prevalent in ageing men and is associated with progressive lower urinary tract symptoms (LUTS), including a slow urinary stream, hesitancy and post-void residual (PVR) [3]. AUR represents one of its most significant complications, with incidence rates depending on age and LUTS severity [3]. Among men over 75 with moderate to severe LUTS, approximately 1 in 3 will experience AUR within the next decade [4].

Standard management of AUR consists of immediate bladder decompression by transurethral catheterization, initiation of alpha-blocker therapy, and a subsequent trial without catheter (TWOC) to assess spontaneous voiding, as recommended by major international guidelines including those of the American Urological Association (AUA) and the National Institute for Health and Care Excellence (NICE) [5–9]. The beneficial effect of alpha-blocker therapy on TWOC success is well-established across multiple systematic reviews [10–12].

Despite widespread adoption of this pathway, the optimal duration of bladder drainage prior to TWOC remains a matter of debate. Although the pharmacokinetics of alpha-blockers, which reach maximum plasma concentrations within 72 hours [13], provide a theoretical rationale for a minimum drainage period, clinical evidence has not consistently translated this into a clear recommendation. Immediate catheter removal is generally avoided, as early recurrence rates of 50% within one week have been reported [14]. Whilst early randomized trials suggested that short-term continued bladder drainage (24 hours to 7 days) improves spontaneous voiding rates compared with immediate catheter removal [15,16], more recent trials incorporating routine alpha-blocker use report only modest differences in TWOC outcomes between day 3 and day 7 [17,18]. Two systematic reviews addressing this question concluded that the overall quality of existing evidence remains insufficient to provide a definitive answer [19,20].

This evidence gap is reflected in current clinical practice: of five major guidelines, only two describe a suggested catheter duration of 48–72 hours, both based on expert opinion rather than robust evidence [5,8]. A recent nationwide Dutch study illustrated de resulting practice variation, with median catheter durations across 13 hospitals ranging from 3 to 18 days and an overall median of 15 days [21].

Such variation carries direct clinical consequences, as catheter‑related morbidity, including catheter-associated urinary tract infections (CAUTIs), hematuria, pain, urethral trauma, and even sepsis, increases with longer catheterization [22–24]. These complications diminish quality of life, increase healthcare utilization, and may even contribute to the broader problem of antimicrobial resistance. Any potential benefit of prolonged bladder drainage on TWOC success must therefore be well balanced against the well-established risks of prolonged catheterization.

Taken together, the available literature and current clinical practice underscore a persistent knowledge gap regarding optimal catheter duration after an episode of AUR [25]. The current study directly addresses this gap by evaluating two catheterization intervals already applied in daily practice – 3 days and 14 days – to determine which approach yields the most favorable balance of voiding outcomes, catheter-related morbidity, patient quality of life, and healthcare resource use. The resulting evidence is intended to refine guidelines, reduce unwarranted practice variation, and ultimately result in cost-effective, evidence-based care for men experiencing AUR.

## 2. Methods

A pragmatic mixed-methods study will be conducted, consisting of a quantitative randomized controlled inferiority trial (RCT) in parallel with a qualitative process evaluation. The RCT (part 1) is designed to assess whether early TWOC at day 3 is non-inferior to delayed TWOC at day 14 among men presenting with AUR, with respect to clinical outcomes, patient burden, and healthcare costs. In the qualitative process evaluation (part 2), we aim to evaluate the experiences and expectations of healthcare professionals regarding both catheter strategies in a qualitative manner.

The current protocol was developed in accordance with the Standard Protocol Items: Recommendations for Interventional Trials (SPIRIT) 2013 guidelines [26] (S1 File).

### 2.1. RCT

#### 2.1.1. Study design.

The pragmatic, multicenter, two-arm randomized controlled non-inferiority study compares early TWOC at 3 days with delayed TWOC at 14 days in men presenting with AUR. The 3-day catheterization period was chosen following recommendations in guidelines [5,8] supported by the pharmacokinetic principle that that alpha-blockers typically reach maximum concentrations within several days [11]. The 14-day catheterization period aligns closely with the median duration observed in Dutch clinical practice of 15 days [21].

#### 2.1.2. Study Setting.

The trial is conducted in at least 11 Dutch academic and non-academic institutions. Eligible patients are primarily identified in emergency departments, general practitioner (GP) practices or out-of-hours GP services, and urology wards, where initial catheterization is performed and alpha-blocker therapy is initiated as part of standard therapy.

#### 2.1.3. Eligibility criteria.

Adult men diagnosed with AUR (retention volume <1500 mL) are eligible for inclusion. All participants must have undergone successful transurethral catheterization and initiated (or continued) alpha-blocker therapy as part of standard care [5,6]. BOO-related AUR is clinically defined as the acute inability to void requiring catheterization, without suspicion of alternative causes (e.g., bladder stones, clot retention, previously diagnosed advanced prostate carcinoma). Imaging or urodynamic confirmation is not required. Detailed inclusion and exclusion criteria are presented in Table 1.

**Table 1:** In- and exclusion criteria for the RELIEF trial.

Inclusion criteria	Exclusion criteria
◦ Male, aged ≥18 years ◦ Presentation with an episode of AUR attributed to suspected BPO ◦ Successful bladder decompression through transurethral catheterization ◦ Initiation or continuation of alpha-blocker therapy at the time of presentation ◦ Ability to adhere to study procedures and provide informed consent	◦ Retention volume > 1500 mL ◦ AUR attributed to suspected: - urethral stricture - bladder stones - clot retention - neurogenic bladder dysfunction ◦ Post-operative AUR, occurring within 72 hours after surgery ◦ History of prostate, urethral, or bladder surgery, except prior TURBT ◦ History of prostate cancer (ISUP grade group ≥2) ◦ Prior episode of AUR within the preceding 30 days ◦ Active bladder cancer or ongoing surveillance for bladder cancer ◦ UTI with fever or urosepsis at the time of presentation

*AUR = acute urinary retention; BPO = benign prostatic obstruction; ISUP = International Society of Urological Pathology grade; TURBT = transurethral resection of bladder tumor; UCC = urothelial cell carcinoma; UTI = urinary tract infection.*

#### 2.1.4. Study objectives.

The primary objective of the RCT is to assess whether an early TWOC (day 3) achieves a success rate that is non-inferior to that of a delayed TWOC (day 14).

Secondary objectives include:

(1) Evaluation of process-related clinical outcomes, including recurrent AUR/ recatheterization rates, and catheter-related complications, such as CAUTIs, hematuria, and urethral strictures.
(2) Evaluation of patient-reported outcomes, including catheter-related burden, LUTS, and health-related quality of life.
(3) Evaluation of resource-related outcomes, including healthcare utilization, travel costs and productivity losses to determine the cost-effectiveness of early versus delayed TWOC.

We hypothesize that early TWOC is non-inferior in terms of restoring spontaneous voiding, while reducing catheter-related morbidity, patient burden, and healthcare costs.

#### 2.1.5. Recruitment and Informed Consent.

Participants will be recruited at the emergency department or urology outpatient clinics of participating hospitals. Patients catheterized by GPs or out-of-hours GP services may also be included, provided that both the time of catheter insertion and the initiation or continuation of alpha-blocker therapy are documented and fall within the specified time window for early TWOC. These patients will be referred to the hospital for a TWOC and will receive subsequent follow-up if indicated.

Informed consent is obtained by the coordinating investigator or a delegated investigator at the local site. To facilitate efficient inclusion, participating centers are supported with standardized study materials, including a dedicated website (www.reliefstudie.com) and a brief informational video outlining the study rationale and procedures. These resources aim to enhance patient understanding and streamline the consent process. Eligible patients receive verbal and written information, including the digital patient information form. They are given at least 24 hours to consider participation before providing informed consent. Consent may be given by returning a signed paper form or digitally via REDCap (Research Electronic Data Capture; Vanderbilt University, Nashville, TN, USA), a secure web-based platform. All forms are countersigned by an investigator prior to randomization.

#### 2.1.6. Randomization and allocation.

After confirming eligibility and obtaining consent, participants are randomized in a 1:1 ratio to either early TWOC (day 3) or delayed TWOC (day 14). Randomization is stratified by study site and performed using a centralized, web-based system. Randomization lists will be computer-generated using permuted block randomization with varying block sizes to prevent predictability of allocation sequences. Allocation concealment is ensured as clinical staff cannot access allocation information prior to randomization.

Blinding of participants and clinicians is not feasible due to the nature of the intervention. This may introduce performance and detection bias; however, bias is minimized by using objective, standardized endpoints and blinded outcome assessors.

#### 2.1.7. Study procedures.

**Fig 1:**
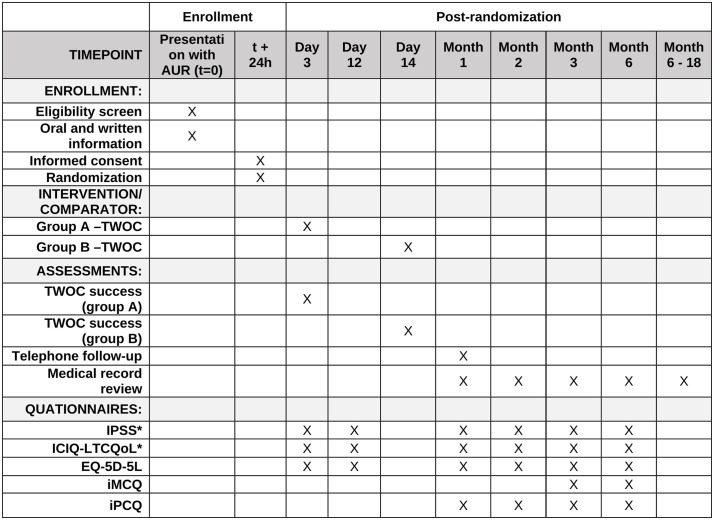
Participant timeline: schedule of enrollment, interventions, and assessments. *Patients with a catheter in situ at the time of assessment complete the ICIQ-LTCQoL, whereas patients without a catheter complete the IPSS. AUR: acute urinary retention; TWOC: trial without catheter; IPSS: International Prostate Symptom Score; ICIQ-LTCQoL: International Consultation on Incontinence Questionnaire – Long-Term Catheter Quality of Life; EQ-5D-5L: EuroQol 5-Dimension 5-Level questionnaire; iMCQ: iMTA Medical Consumption Questionnaire; iPCQ: iMTA Productivity Cost Questionnaire; PVR: post-void residual.

All participants will undergo a TWOC in a clinical or outpatient setting to ensure accurate collection of primary outcome data. The allocated catheter duration is followed strictly on working days; if the scheduled TWOC date falls on a weekend, it is performed on the next working day. A schematic overview of the study flow is provided in Fig 1.

Participating centers organize their own TWOC appointments and may perform the procedure either on the ward or in an outpatient setting, in line with local practice patterns. All sites will document the voided volume and measure the post-void residual within 30 minutes after micturition using bladder ultrasound. This workflow preserves the pragmatic nature of the study, while ensuring that the key outcome measures are collected in a consistent and comparable manner across sites.

TWOC success is determined according to the study’s predefined criteria. Decisions regarding subsequent recatheterization are left to the treating clinician and may vary across sites and individual cases. Additional diagnostic evaluations (e.g., digital rectal examination, urodynamic assessment, prostate imaging) and further therapeutic steps are likewise performed at the discretion of the local clinical team.

Procedural and clinical characteristics, including TWOC location, bladder filling, flowmetry, number of voiding attempts, recatheterization during the TWOC, and follow-up management, are recorded systematically for each participant throughout the trial. The pragmatic approach of allowing site-level procedural variation while standardizing outcome assessment enhances the clinical generalizability of the findings to routine urological practice.

#### 2.1.8. Outcome measures.

*Primary outcome:* The primary outcome is the success of the TWOC, defined as restoration of spontaneous voiding, immediately after catheter withdrawal, with a voided volume ≥100 mL and a PVR ≤ 200 mL.

*Secondary outcomes:* Secondary outcomes are grouped into three domains. Measurement methods and timing are outlined per domain and summarized in Table 2.

**Table 2:** Outcome Measures and Assessment Schedule.

Domain	Outcome Measure	Day 3	Day 12	Month 1	Month 2	Month 3	Month 6	≤30 days post-TWOC	≤18 months post-TWOC
**Patient-reported outcomes**	IPSS*	✔	✔	✔	✔	✔	✔		
	ICIQ-LTCQoL**	✔	✔	✔	✔	✔	✔		
	EQ-5D-5L	✔	✔	✔	✔	✔	✔		
**Process-related outcomes**	Short-term complications (CAUTI, hematuria, pain, recatheterization, urosepsis)							✔	
	Long-term complications (e.g., urethral stricture)								✔
**Resource-use outcomes**	iPCQ			✔	✔	✔	✔		
	iMCQ					✔	✔		
	Follow-up healthcare use								✔

* The IPSS is administered only to participants who are not catheterized at the time of assessment.

** The ICIQ-LTCqoL is administered only to participants who have an indwelling catheter at the time of assessment.

*TWOC: trial without catheter; IPSS: International Prostate Symptom Score; ICIQ-LTCQoL: International Consultation on Incontinence Questionnaire – Long-Term Catheter Quality of Life; EQ-5D-5L: EuroQol 5-Dimension 5-Level questionnaire; iMCQ: iMTA Medical Consumption Questionnaire; iPCQ: iMTA Productivity Cost Questionnaire.*

A. Process-related clinical outcomes:
  ◦ Recurrent retention requiring recatheterization within 30 days after TWOC;
  ◦ Short-term complications, including CAUTI, urosepsis, macroscopic hematuria, and catheter-related discomfort, are captured through review of clinical records and a telephone assessment approximately 30 days after TWOC.
  ◦ Long-term complications and urological outcomes, primarily the development of symptomatic urethral strictures, are documented through medical record review up to 18 months.

All complications will be systematically assessed and graded according to the Clavien-Dindo classification [27].

B. Patient-reported outcomes:
  ◦ LUTS as measured by the International Prostate Symptom Score (IPSS) [28]
  ◦ Catheter-related symptoms and burden as measured by the International Consultation on Incontinence Questionnaire - Long-Term Catheter Quality of Life (ICIQ-LTCqoL) [29]
  ◦ General health-related quality of life: EuroQol 5-Dimension 5-Level questionnaire (EQ-5D-5L) [30]

Patient-reported assessments are conducted at predefined intervals during the first six months, as detailed in Table 2. At every assessment, participants will fill out patient-reported outcome measures (PROMs). Whether the IPSS or ICIQ-LTCqol is administered depends on whether the participant has an indwelling catheter at the time of assessment. When a participant does not have an indwelling catheter at the time of assessment, the IPSS will be completed. When a participant does have an indwelling catheter, the ICIQ-LTCqoL will be completed.

C. Resource-related outcomes:

Healthcare utilization related to the assigned catheterization duration, including catheter replacements, GP or outpatient consultations, emergency department visits, hospital admissions, and treatment of short-term complications, is primarily captured through medical record review and a telephone assessment approximately 30 days after TWOC. Long-term healthcare utilization, including diagnostic procedures, any treatment for BPH during follow-up and the treatment of long-term complications, is captured through medical record review throughout the 18-month follow-up period. As this data collection method is limited to in-hospital care from 30 days post-TWOC, it is supplemented by the iMTA Medical Consumption Questionnaire (iMCQ) to capture out-of-hospital healthcare utilization beyond this period [31]. Productivity losses are assessed using the iMTA Productivity Cost Questionnaire (iPCQ) [32]. Both questionnaires are administered at prespecified intervals up to 6 months (Table 2).

To promote questionnaire completion, reminders are sent via Research Manager.

#### 2.1.9. Sample size calculation.

The sample size calculation is based on the non-inferiority design comparing the success rates of early and delayed TWOC. Non-inferiority is defined as an absolute difference in success rates not exceeding a margin of 12%, based on both prior literature and consensus among all stakeholders of the study [19,20]. This margin was established by weighing the risks of an unsuccessful TWOC requiring recatheterization (a common procedure that, whilst burdensome, carries limited long-term consequences) against those of catheter-related complications (including CAUTIs sepsis and bladder spasms), which impose a substantial burden on patients, healthcare systems, and broader public health. Stakeholders agreed that early catheter removal would remain a clinically preferable strategy provided its TWOC success rate did not fall more than 12% below that of delayed removal, reflecting the acceptable trade-off between the competing risks.

Assuming a re-catheterization rate of approximately 50%, a total of 478 participants (239 per arm) is required to achieve 80% power at a one-sided significance level of 5% (α = 0.05) [23,24]. This sample size accounts for an anticipated dropout rate of 10% during the trial.

This sample size provides adequate precision to evaluate whether shortened catheterization maintains comparable clinical effectiveness while potentially reducing catheter-related burden and enhancing patient comfort. No interim analysis is planned, as repeated testing would risk inflation of the type I error rate.

#### 2.1.10. Data Management.

The handling of personal data complies with European General Data Protection Regulation (GDPR) and the Dutch Act on Implementation of the General Data Protection Regulation (in Dutch: Uitvoeringswet AVG).

All study data are entered into electronic case report forms (eCRFs) using Research Manager (Cloud9 Software, Deventer, the Netherlands); a validated, Good Clinical Practice (GCP)-compliant platform with role-based access control. Data will be pseudonymized by assigning each participant a unique study ID. Access to the database will be restricted to authorized study staff and monitored through personal login credentials. Patient identifiers will be stored separately from the research data. Pseudonymized data will be retained for 15 years after study follow-up ends. Data integrity is promoted through audit trails, and periodic monitoring of data completeness and accuracy.

*Monitoring and inspection:* Monitoring is coordinated by the sponsor and carried out by a qualified monitor independent from the study team, verifying protocol adherence, data quality, and compliance with regulatory requirements. Monitoring visits will be conducted at the beginning and end of the trial, with at least one visit per year. Frequency may be adjusted based on inclusion rate and previously observed deviations. The study may additionally be subject to audit by the sponsor institution or external regulatory bodies to ensure compliance with GCP and applicable GDPR.

#### 2.1.11. Ethical Considerations.

*Patient Burden and Risk-Benefit Assessment:* This study poses minimal risks to participants, as both catheterization strategies under investigation are conducted in current clinical practice and constitute standard care for AUR. Participants in both study arms receive alpha-blocker therapy and attend the urology outpatient department or ward for catheter removal and TWOC assessment, in line with usual care. No additional clinical procedures are mandated beyond the timing of catheter removal. Study-related burden is limited to online questionnaire completion at six predefined time points within the first six months, and one telephone assessment, minimizing extra visits or tests. Participants receive a €30 gift voucher as compensation for their time and completion of the study-related questionnaires.

Overall, the anticipated risks and benefits are considered ethically appropriate for research participation.

*Ethics approval:* The study was approved by the Medical Ethics Review Committee (METc) of the University Medical Center Groningen (UMCG) (METc no. 2025/185), as well as by the local Ethical Review Boards of all participating sites. Protocol revisions will be communicated to relevant parties.

#### 2.1.12. Safety and Adverse Events.

Safety monitoring will comply with the Dutch Medical Research Involving Human Subjects Act. All adverse events (AEs) related to the catheter, early TWOC, or subsequent re-catheterization will be documented for later analysis. Only serious adverse events (SAEs) deemed related to the study intervention will be reported immediately to the sponsor and the accredited ethics committee. Life-threatening or fatal SAEs will be reported within 7 days; other SAEs within 15 days. Unrelated SAEs will not be reported, as no patient benefit is anticipated.

Safety data will be actively monitored throughout the trial. If safety issues are identified, the sponsor may decide to suspend the trial and will then promptly inform the accredited ethics committee.

#### 2.1.13. Sponsor and Trial Registration.

Isala acts as the sponsor of this trial. Specific responsibilities may be delegated to participating hospitals, as appropriate.

The trial is registered at ClinicalTrials.gov (identifier: NCT07283484), as well as in the Dutch national research registry (Overzicht van Medisch-wetenschappelijk Onderzoek in Nederland; NL-009284).

#### 2.1.14. Patient involvement.

Patients were involved during the development of this trial, including review of the participant information form and assessment of study burden. One of the PROMs used in this trial, the ICIQ-LTCqol, was translated into Dutch and validated in parallel to this study; during this process, interviews with catheter users were conducted to assess the comprehensibility and relevance of the questionnaire items. Patients will be involved in the dissemination of findings upon completion of this trial, including a plain language summary.

#### 2.1.15. Trials status.

The trial is currently in the recruitment phase. Recruitment commenced on 21/08/2025 and is expected to be completed within two-and-a-half years. Data collection for the primary outcome and short-term complications will be completed within 30 days after the last participant is enrolled. Results regarding long-term complications will be available 18 months after the last participant is enrolled. Primary results are expected to be submitted for publication approximately three years after recruitment initiation.

### 2.2. Process evaluation

#### 2.2.1. Aim.

To complement the quantitative findings of the RCT, a qualitative process evaluation will be conducted to explore healthcare professionals’ perspectives on the implementation of a standardized TWOC strategy following AUR in men. This sub-study aims to identify barriers and facilitators shaping current catheterization practices, and to assess the feasibility, acceptability, and sustainability of early versus delayed TWOC strategies within clinical practice.

#### 2.2.2. Design and Participants.

A purposive sampling strategy will be used to recruit healthcare professionals directly involved in the management or planning of AUR care within hospitals participating in the RELIEF trial. Eligible participants include urologists, residents, non-training residents, nurse specialists, nurses, and planning staff. Recruitment will continue until thematic saturation is achieved, expected at 9–17 participants [33].

#### 2.2.3. Data collection.

Semi-structured interviews will be conducted at two stages:

(1) prior to RCT initiation, focusing on perceived drivers of current practice variation in TWOC planning, and anticipated barriers and facilitators for implementing standardized TWOC strategies within the context of the trial;
(2) during conduction of the RCT, focusing on actual experiences with implementation of standardized TWOC strategies within the context of the trial, and anticipated barriers and facilitators for implementing standardized TWOC strategies within future clinical practice.

Interviews will be held face-to-face, by telephone, or via secure video conferencing, based on participant preference, and will last approximately 15–45 minutes. All interviews will be audio-recorded and transcribed verbatim. A semi-structured interview guide informed by the Consolidated Framework for Implementation Research (CFIR) will structure the dialogues, thereby ensuring the systematic exploration of key domains while allowing space for unanticipated issues to emerge [34]. The guide may be refined iteratively during data collection to incorporate novel insights.

#### 2.2.4. Data analysis.

Transcripts will be analyzed inductively using Braun and Clarke’s six-phase thematic analysis approach, supported by Atlas.ti® qualitative data analysis software (ATLAS.ti Scientific Software Development GmbH), including its integrated Intentional Artificial Intelligence (IAI) tool. The primary researcher will conduct the initial coding of all transcripts. AI-generated suggestions will be critically reviewed and refined to ensure accurate representation of the data. To enhance the reliability of the analysis, a second researcher will independently review and code a subset of transcripts, after which discrepancies will be resolved through discussion.

A constant comparative method will be employed to identify both shared and divergent perspectives across participants. Reflexivity will be maintained throughout the analysis to enhance transparency and to limit the influence of potential researcher bias.

#### 2.2.5. Ethical Considerations.

This qualitative study adheres to the Consolidated Criteria for Reporting Qualitative Research (COREQ) guidelines for reporting qualitative research and follows institutional policies on ethical conduct and data protection [35]. Findings from the qualitative sub-study will be reported separately. While formal integration of the qualitative and quantitative components is beyond the scope of this protocol, quantitative findings, for example procedural predictors of TWOC success, are expected to support the future implementation of a standardized TWOC strategy in clinical practice.

## 3. Results

### 3.1. Participant Flow

Participant flow, including screening, enrollment, randomization, and follow-up will be reported in accordance with CONSORT 2025 guidelines [36]. A CONSORT flow diagram will be presented upon completion of the trial, outlining the number of participants at each stage and the reasons for exclusion or dropout.

### 3.2. Baseline Characteristics Assessment

Baseline demographic and clinical characteristics will be summarized by study arm using descriptive statistics. Variables will include age, comorbidities, retention volume at presentation, prior and current alpha-blocker use, prostate volume (if available), and the setting in which catheter placement and removal occurred. No formal statistical testing of baseline differences will be performed, consistent with CONSORT recommendations; instead, baseline data will be presented descriptively to facilitate clinical interpretation of group comparability.

### 3.3. Primary Outcome

The primary outcome (TWOC success) will be analyzed primarily using a per-protocol approach, with a complementary intention-to-treat analysis to confirm non-inferiority. The difference in TWOC-success proportions between the early and delayed TWOC groups will be estimated with a one-sided 95% confidence interval. Non-inferiority of early TWOC will be concluded if this confidence interval lies entirely within the pre-specified 12% non-inferiority margin. Because data will be collected across multiple institutions, a multilevel (mixed effects) analysis will be performed to account for clustering of patients within institutions.

### 3.4. Secondary outcomes

Secondary outcomes will be analyzed according to their data type and timing.

◦ Process-related clinical outcomes: catheter- and TWOC-related complications will be reported descriptively and compared between groups using appropriate statistical tests for categorical data (e.g., Fisher’s exact test).
◦ Patient-reported outcomes**:** scores from the IPSS, ICIQ-LTCqoL, and EQ-5D-5L will be analyzed using longitudinal data analysis to evaluate differences in outcome trajectories between groups.
◦ Resource-related outcomes: reported healthcare use and productivity losses, supplemented with the results of the EQ-5D-5L, will serve as input for the trial-based economic evaluation described below.

Cost-effectiveness analysis: A trial-based economic evaluation will be conducted from a healthcare and a societal perspective over an 18-month follow-up period, using a template code developed by Ben et al [37]. Analyses will adhere to Dutch guidelines for economic evaluations in healthcare [38]. Costs will be calculated through multiplying resource use with the accompanying unit costs. Quality-adjusted life years (QALYs) will be calculated from EQ-5D-5L data by first converting each reported health-state level into a utility value using the Dutch value set by Versteegh et al. (2016) [39]. These utility values are then multiplied with the time between the follow-up assessments to derive QALYs. Missing data will be multiply-imputed using the *mice* package in R [40]. Costs will be discounted at 3.0% per year and QALYs at 1.5% per year [38]. Incremental cost-effectiveness ratios (ICERs) will be estimated using non-parametric bootstrapping combined with seemingly unrelated regressions and presented on a cost-utility plane and cost-effectiveness acceptability curves.

### 3.5. Multivariable logistic regression

To identify predictors of TWOC success, a multivariable logistic regression analysis will be performed. Pre-specified variables include age, retention volume, prostate volume (if known), whether alpha-blocker therapy was initiated or continued, alcohol intake prior to the AUR episode, bladder filling volume prior to TWOC, and number of voiding attempts.

Results will be reported as adjusted odds ratios with 95% confidence intervals. All analyses will be considered exploratory, as the trial is not powered to detect predictor-specific effects.

### 3.6. Missing data

Given the prospective design of the study and the short interval between randomization and TWOC, missing data for TWOC success are expected to be minimal. For secondary outcomes, missingness patterns will be explored by comparing participants with and without missing data on observed baseline and follow-up variables. Where appropriate, missing data will be handled using multiple imputation by chained equations [40]. This strategy will generate imputed datasets, with results subsequently pooled using Rubin’s rules to ensure reliable analysis [41]. Sensitivity analyses will be conducted to assess the impact of missing data on the overall results.

### 3.7. Dissemination plans

The investigators intend to publish the results of this trial in a peer-reviewed journal and present them at national and international conferences, regardless of the outcome. Trial results will be reported in the OMON registry. A plain language summary will be made available to participants and the public upon completion of the trial.

## 4. Discussion

The RELIEF trial is initiated in response to the long-standing evidence gap regarding the optimal duration of catheterization following AUR [25]. It incorporates several design choices that aim to address limitations of prior work. Alpha-blocker therapy is standardized across all participants, and TWOC success is defined using objective, volume-based criteria consistent with current urological practice. In addition to immediate voiding outcomes, the trial systematically captures short- and long-term complications as well as patient-reported outcomes, including LUTS severity, healthcare consumption, productivity losses, catheter-related burden and health-related quality of life. This comprehensive approach aligns with earlier calls to incorporate broader, patient-centered endpoints in AUR research [24].

The non-inferiority design is well suited to this research question, as early TWOC is intended to lessen catheter-related morbidity and associated patient and healthcare burden rather than to increase TWOC success itself. Within this design, the selected intervention time points are clinically and pharmacologically justified. The 3-day time point is based on pharmacokinetic data showing that alpha-blockers typically reach steady-state concentrations within several days, whereas the 14-day comparator reflects the median catheter duration of 15 days (IQR 6–23 days), observed in Dutch clinical practice [21]. To define an acceptable balance between effectiveness, tolerability, and safety, the non-inferiority margin was set at 12 percentage points. This margin was informed by the variability in TWOC success rates reported in earlier studies [19,20] and supported by expert consensus among all stakeholders and reviewers of the protocol in the grant submission phase. It represents the threshold beyond which early removal may no longer be considered an acceptable alternative, balancing clinical acceptability with statistical feasibility. Stratification by center and objective endpoint definitions further enhance internal validity, whilst inclusion of diverse hospital types (academic vs non-academic) and different regions in the Netherlands support external validity.

Several limitations warrant consideration. Variation in TWOC procedures across centers, including decisions regarding bladder filling, the number of voiding attempts, and the clinician’s choice for recatheterization, may introduce heterogeneity. However, these procedural differences are systematically recorded and reflect clinical practice, thereby supporting generalizability of the findings. In addition, their potential impact is minimized by block randomization across centers.

Deviations from the allocated TWOC timing may occur in either study group. Their impact will be evaluated by conducting both per-protocol and intention-to-treat analyses, supplemented where necessary by sensitivity analyses to assess the robustness of the findings. Lastly, as an open-label trial, some degree of performance or detection bias cannot be fully excluded, although the objective nature of the primary outcome is expected to mitigate this risk.

The inclusion of both a health economic evaluation and a qualitative process evaluation strengthens the trial’s capacity to implement its findings into practice, irrespective of the eventual outcomes. The health economic component is expected to generate reliable estimates of the cost-effectiveness of early versus delayed TWOC, whilst the qualitative component may identify organizational and behavioral factors that influence the feasibility and sustainability of implementing a standardized TWOC strategy. Together, these elements complement existing registry and survey data that have documented practice variation, while leaving the underlying mechanisms of such variation unexplored [21,23,24].

Taken together, the RELIEF trial is designed to generate high-quality evidence on the optimal catheter duration prior to TWOC. By combining clinical, economic, and implementation perspectives, the study is expected to provide an evidence-based foundation for future guideline refinement, resulting in more uniform, cost-effective and patient-centered care for men experiencing AUR.

## Supporting information

S1 File SPIRIT checklist. (PDF)

S2 File Approved study protocol. (PDF)

## Abbreviations

AE: adverse event
AUR: acute urinary retention
BPH: benign prostatic hyperplasia
BOO: bladder outlet obstruction
CAUTI: catheter-associated urinary tract infection
EAU: European Association of Urology
EQ-5D-5L: EuroQol 5-Dimension 5-Level questionnaire
GDPR: General Data Protection Regulation
GP: general practitioner
ICIQ-LTCqoL: International Consultation on Incontinence Questionnaire–Long-Term Catheter Quality of Life;
IPSS: International Prostate Symptom Score
iMCQ: iMTA Medical Consumption Questionnaire
iPCQ: iMTA Productivity Cost Questionnaire
LUTS: lower urinary tract symptoms
PVR: post-void residual
SAE: serious adverse event
TWOC: trial without catheter.
