# Supplementary material for: Early versus delayed trial without catheter in men with Acute urinary retention - Study Protocol for a dutch national randomized trial
Source: PLoS One. 2026 Aug 6;21(8):e0354879. doi: 10.1371/journal.pone.0354879 (PMC13446707; doi:10.1371/journal.pone.0354879)
Supplement: Supplementary file 2 [file pone.0354879.s002.pdf]

RESEARCH PROTOCOL

RELIEF STUDY

(May 2025)

PROTOCOL TITLE '*Early Versus Delayed Trial Without Catheter in Men with Acute Urinary Retention: a Randomized Controlled Trial (the RELIEF study).'*

**Protocol ID**	NL-009284
**Short title**	RELIEF study
**Version**	1.1
**Date**	19-05-2025
**Coordinating investigator/project leaders**	Coordinating investigator:<br>Drs. L.L.A. Ribbert, Isala, afdeling Urologie<br>Project leaders:<br>Dr. L.P.W. Witte, Isala, afdeling Urologie<br>Prof. dr. M.H. Blanker, UMCG, afdeling Huisartsgeneeskunde en Ouderengeneeskunde
**Principal investigator(s) (in Dutch: hoofdonderzoeker/ uitvoerder)**	Isala – Dr. L.P.W. Witte<br><br>Alrijne Ziekenhuis – Dr. B.M.A. Schout<br>Catharina Ziekenhuis – Dr. E.L. Koldewijn<br>Canisius Wilhelmina Ziekenhuis – Dr. D. Duijvesz<br>Martini Ziekenhuis – Dr. J.G.H. Poerink<br>Maastricht UMC - Prof. dr. J.P.F.A. Heesakkers<br>Onze Lieve Vrouwe Gasthuis – Dr. E.P. van Haarst<br>Spaarne Gasthuis – Dr. L. Gietelink<br>St. Jansdal – Dr. C. ten Donkelaar<br>Ziekenhuisgroep Twente – Dr. C.A.M. van der Fels<br>Zuyderland Ziekenhuis – Dr. M. Bruins
**Sponsor (in Dutch: verrichter/opdrachtgever)**	Isala, afdeling Urologie
**Subsidising party**	ZonMw

**Independent expert(s)**	Dr. M.K. Engberts, Isala, afdeling Gynaecologie

TABLE OF CONTENTS

1. INTRODUCTION AND RATIONALE ... 9
2. OBJECTIVES ... 11
3. STUDY DESIGN ... 12
4. STUDY POPULATION ... 15
4.1 Population (base) ... 15
4.2 Inclusion criteria ... 15
4.3 Exclusion criteria ... 15
4.4 Sample size calculation ... 15
5. TREATMENT OF RESEARCH PARTICIPANTS ... 16
5.1 Investigational product/treatment ... 16
5.2 Use of co-intervention (if applicable) ... 16
5.3 Escape medication (if applicable) ... 16
6. METHODS ... 17
6.1 Study parameters/endpoints ... 17
6.1.1 Main study parameter/endpoint ... 17
6.1.2 Secondary study parameters/endpoints (if applicable) ... 17
6.1.3 Other study parameters ... 17
6.2 Randomization, blinding and treatment allocation ... 18
6.3 Study procedures ... 18
6.3.1 Catheter withdrawal ... 18
6.3.2 Follow-up ... 18
6.4 Withdrawal of individual research participants ... 19
6.5 Replacement of individual research participants after withdrawal ... 20
6.6 Follow-up of research participants withdrawn from treatment ... 20
6.7 Premature termination of the study ... 20
7. SAFETY REPORTING ... 21
7.1 Temporary halt for reasons of research participant safety ... 21
7.2 AEs, SAEs ... 21
7.2.1 Adverse events (AEs) ... 21
7.2.2 Serious adverse events (SAEs) ... 21
7.3 Follow-up of adverse events ... 22
7.4 Data Safety Monitoring Board (DSMB) / Safety Committee ... 22
8. STATISTICAL ANALYSIS ... 23
8.1 Primary study parameter(s) ... 23
8.2 Secondary study parameter(s) ... 23
8.3 Other study parameters ... 24
8.4 Interim analysis (if applicable) ... 24
9. ETHICAL CONSIDERATIONS ... 25
9.1 Regulation statement ... 25
9.2 Recruitment and consent ... 25

9.2.1 Recruitment 25
9.2.2 Informed consent procedures 25
9.2.3 Digital informed consent 26
9.3 Benefits and risks assessment, group relatedness 26
9.4 Compensation for injury 27
9.5 Incentives (if applicable) 27
10. ADMINISTRATIVE ASPECTS, MONITORING AND PUBLICATION 28
10.1 Handling and storage of data and documents 28
10.2 Monitoring and Quality Assurance 28
10.3 Amendments 29
10.4 Annual progress report 29
10.5 Temporary halt and (prematurely) end of study report 29
10.6 Public disclosure and publication policy 29
11. REFERENCES 30

LIST OF ABBREVIATIONS AND RELEVANT DEFINITIONS

**AE**	**Adverse Event**
**AUR**	**Acute Urinary Retention**
**CCMO**	**Central Committee on Research Involving Human Subjects; in Dutch: Centrale Commissie Mensgebonden Onderzoek**
**DM**	**Diabetes Mellitus**
**DRE**	**Digital Rectal Examination**
**DSMB**	**Data Safety Monitoring Board**
**eCRF**	**Electronic Case Report Form**
**GCP**	**Good Clinical Practice**
**GDPR**	**General Data Protection Regulation; in Dutch: Algemene Verordening Gegevensbescherming (AVG)**
**ICF**	**Informed Consent Form**
**ICIQ**	**International Consultation on Incontinence Questionnaires**
**IPSS**	**International Prostate Symptom Score**
**METC**	**Medical research ethics committee (MREC); in Dutch: medisch-ethische toetsingscommissie (METC)**
**PV**	**Prostate Volume**
**QoL**	**Quality of Life**
**Review committee**	**Medical research ethics committee (MREC) or CCMO**
**(S)AE**	**(Serious) Adverse Event**
**Sponsor**	**The sponsor is the party that commissions the organisation or performance of the research, for example a pharmaceutical company, academic hospital, scientific organisation or investigator. A party that provides funding for a study but does not commission it is not regarded as the sponsor, but referred to as a subsidising party.**
**TUC**	**Transurethral Catheter**
**TWOC**	**Trial Without Catheter**
**UAVG**	**Dutch Act on Implementation of the General Data Protection Regulation; in Dutch: Uitvoeringswet AVG**
**UTI**	**Uninary Tract Infection**
**WMO**	**Medical Research Involving Human Subjects Act; in Dutch: Wet Medisch-wetenschappelijk Onderzoek met Mensen**

SUMMARY

Rationale: Management of acute urinary retention (AUR) with a transurethral catheter (TUC) lacks consensus on the optimal duration. Existing research shows conflicting results, and practice variation among urologists is high. A shorter catheterization period may reduce complications, improve patient comfort, and lower costs, whereas a longer duration may improve success rates of trial without catheter (TWOC). We hypothesize that a 3-day catheter duration is non-inferior to a 14-day duration regarding re-catheterization rates, with the potential for fewer complications and improved quality of life (QoL).

Objectives: Main Objective: To determine whether the re-catheterization rate following a short (3-day) TUC duration is non-inferior to that following a long (14-day) TUC duration in men with acute urinary AUR treated with both TUC and alpha-blockers. Secondary Objectives: To assess differences in patient-centered outcomes (symptoms, QoL), process outcomes (complications), and resource outcomes (healthcare and societal costs) between the two catheter durations.

Study design: Multicenter, randomized, controlled, non-inferiority trial with a 1:1 allocation to either a short or long catheter duration (ZonMw funded, dossiernummer: 10390032310057).

Study population: Adult men with AUR treated with a TUC and alpha-blockers.

Intervention: TWOC 3 days after catheter insertion.

Main study parameters/endpoints: Re-catheterization rate immediately after TWOC (success vs. failure of spontaneous voiding).

Nature and extent of the burden and risks associated with participation, benefit and group relatedness: All participants undergo standard AUR procedures: TUC placement, alpha-blocker therapy, TWOC, and follow-up visits. A shorter catheter duration may reduce the risk of catheter-related infections and discomfort, while maintaining similar re-catheterization rates. However, it may also increase the likelihood of early AUR recurrence. Both groups complete questionnaires on days 3 and 12, and at 1, 2, 3 and 6 months. This study is expected to offer a more efficient, evidence-based approach to AUR management, potentially improving patient well-being and reducing healthcare expenditures.

1. INTRODUCTION AND RATIONALE

Acute urinary retention (AUR) in men is a urological emergency, defined as a sudden and painful inability to pass urine (1). It is widely acknowledged as a serious complication linked to the progression of benign prostatic hyperplasia (BPH). In The Netherlands alone, approximately 40,000 men experience AUR annually. Standard management typically involves the placement of a transurethral catheter (TUC) and the administration of an alpha-blocker, followed by a trial without catheter (TWOC) to evaluate whether patients are able to resume spontaneous micturition. However, the optimal catheter duration prior to TWOC remains undetermined.

Despite its high prevalence, AUR and its management have not been conclusively studied, leading to its identification as a knowledge gap in the Dutch Urological Society (NVU) 2020-2024 agenda (2). Research on catheter duration has yielded conflicting results. Some publications suggest that a longer catheterization period may enhance TWOC success rates, while others find no significant difference (3-10). This inconsistency is reflected in clinical practice, where the duration of catheterization varies significantly. A recent Dutch cross-sectional study (AUR-SNAPSHOT, ZonMw funded, dossiernummer: 10390092110026) reported TUC use ranging from 6 to 24 days (median 15 days), further highlighting the lack of consensus (11).

This uncertainty places a significant burden on patients, as longer catheter use may cause discomfort, elevate the risk of infections, and increase healthcare costs (6,7,8,10,12,13). The AUR-SNAPSHOT study also underscores the need for further investigation into the impact of catheter duration on complications within the AUR population, such as hematuria (12.3%), urinary tract infections (10.3%), and urosepsis (4.3%) (11). These findings serve as a springboard for the RELIEF study, which aims to generate high-quality evidence to fill the existing gaps.

The RELIEF study will focus on adult men presenting with AUR, the primary group affected by BPH-related urinary complications. Evidence suggests that alpha-blocker therapy enhances TWOC success, achieving steady-state levels around day three (14-19). Among five guidelines (20-24), two (20,23) endorse a three-day catheterization period, largely based on expert consensus, while the others do not specify a recommendation on catheter duration.

The absence of conclusive data and definitive guidelines underscores the necessity for a well-designed study to assess whether a shorter catheterization duration (3 days) is non-inferior to a longer duration (14 days) in terms of re-catheterization rates. The RELIEF study will address this gap by systematically evaluating both clinical and patient-centered outcomes, including complications, quality of life (QoL), and healthcare expenditure.

By directing attention to men with AUR, who frequently experience significant pain and discomfort, this research aims to determine the most effective, patient-centered, and cost-efficient catheter duration in a population for which standardized care is urgently needed. Such findings may substantially refine clinical recommendations, reduce practice variation, and ultimately improve the well-being of men with AUR.

2. OBJECTIVES

Primary Objective:

To determine whether the re-catheterization rate after a short catheter duration (3 days) is non-inferior to that after a longer catheter duration (14 days) in men with AUR who are treated with a TUC and alpha-blocker therapy.

This central question dictates the study design and required sample size.

Secondary Objective(s):

- Patient-Centered Outcomes: To compare the impact of a short (3-day) vs. long (14-day) catheter duration on patient-reported symptoms and QoL.
- Resource-Related Outcomes: To evaluate whether a shorter catheter duration reduces healthcare and societal costs compared to a longer duration.
- Process-Related Outcomes: To assess differences in complication rates, such as hematuria, catheter-associated urinary tract infections (CAUTI), or catheter-related discomfort between the two catheter durations.

Hypothesis

It is hypothesized that a 3-day catheter duration will not be inferior to a 14-day catheter duration regarding re-catheterization rates. Furthermore, shorter catheterization is expected to yield fewer symptoms and complications, improved QoL and lower costs compared to longer catheterization in men with AUR.

3. STUDY DESIGN

A multicenter, randomized, controlled, non-inferiority trial will be conducted with a 1:1 allocation (computer-generated, variable block sizes). Due to the variation in catheter durations, blinding of participants and clinicians is not feasible during the trial. However, data analysts will remain blinded to the group assignments of each participant.
A total of 11 Dutch hospitals will be participating.

Justification for the design
We conduct an RCT rather than an observational study, in which all patients would undergo a 3-day TWOC, followed by a 14-day TWOC for those who fail the initial attempt. An observational design would not allow for a direct comparison of primary and secondary outcomes between equivalent groups.
The choice for a 3-day catheterization period is based on the pharmacodynamic profile of alpha-blockers. With daily administration, steady-state plasma levels are typically reached within 2 to 3 days (14-19). This corresponds with the time point at which the maximal therapeutic effect on alpha-1 adrenergic receptors in the bladder, prostate, and urethra is expected - leading to optimal smooth muscle relaxation and improved conditions for a successful TWOC.
A non-inferiority design is the most suitable approach to assess whether a 3-day catheter duration is at least as effective as a 14-day duration in reducing the need for re-catheterization. This design was selected over a superiority design, as we anticipate only a small difference in re-catheterization rates between the two study groups.

Non-inferiority margin
A non-inferiority margin of 12% was selected based on statistical considerations, clinical relevance, and the feasibility of achieving realistic sample sizes in the study arms. Three prior randomized controlled trials reported no significant differences in re-catheterization rates between short and long catheter durations, ranging from 11% to 16.4% (4,5,10). Additionally, the 12% margin was considered appropriate by an expert panel that contributed to the development of the study protocol. The panel discussed that when more frequent re-catheterization in the study group occurs, the associated clinical risks are relatively low, and potential complications are generally mild and manageable. A broader non-inferiority margin is deemed acceptable, as it is expected that the benefits of reduced catheter burden across the entire population, such as fewer complications and improved patient comfort, will outweigh the drawbacks of more frequent re-catheterization in the study group. Furthermore,

the implementation of a strict non-inferiority margin would result in a risk of Type II error, where the intervention might incorrectly be deemed inferior.
Altogether, this margin offers a feasible and robust approach to addressing the primary research question.

Duration and setting

The follow-up period to capture the primary outcome, as well as short-term catheter-related complications and costs will last 6 months. An additional 12 months of medical record screening will be conducted to document any potential interventions for BPH, along with long-term complications (primarily urethral strictures). All TWOC procedures will be conducted in an outpatient or clinical setting.

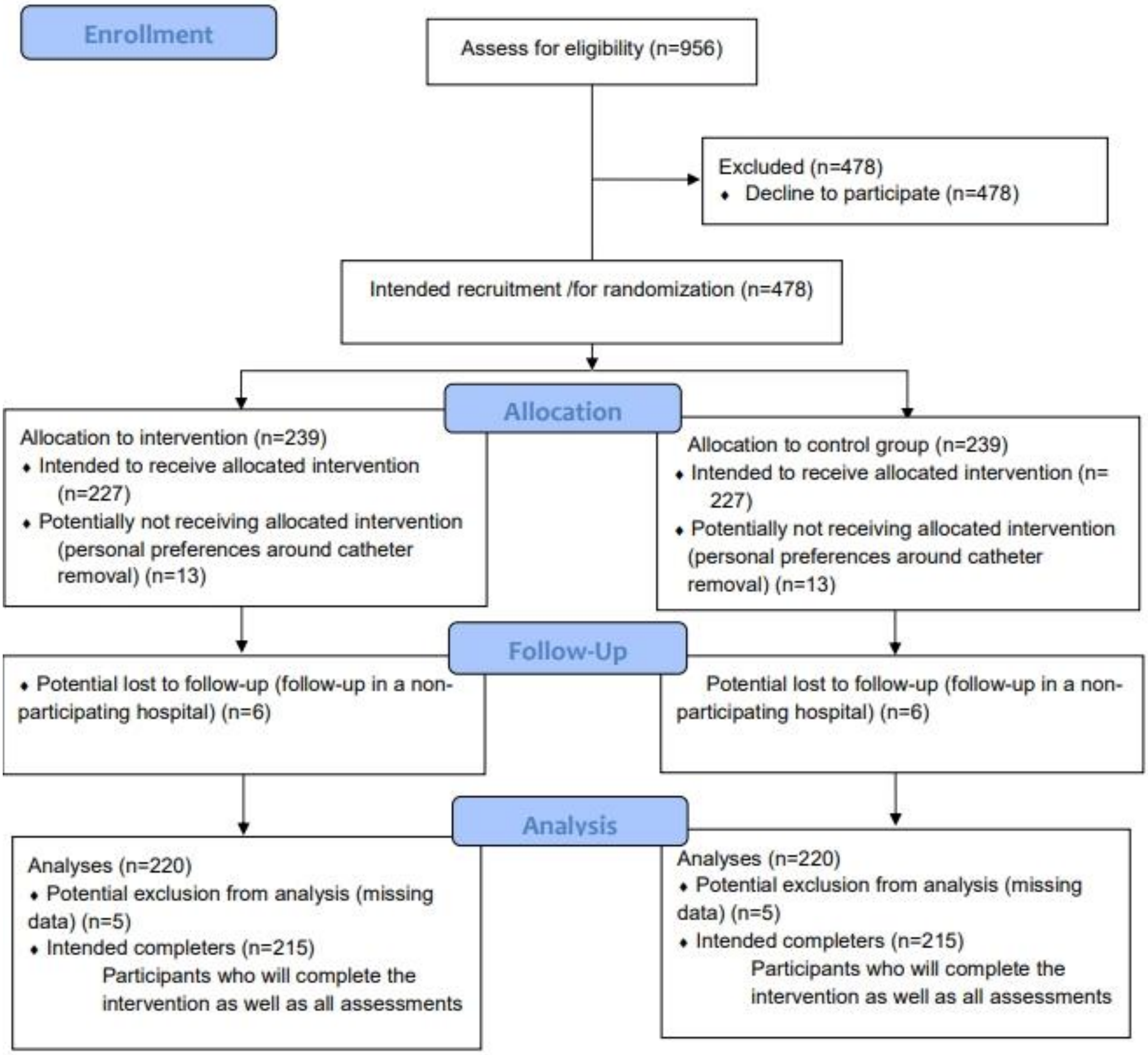

Figure 1: Flow diagram - Patient inclusion

4. STUDY POPULATION

4.1 Population (base)

Adult men diagnosed with AUR and treated with a transurethral catheter (TUC) and alpha-blocker therapy are eligible for inclusion in the study.

4.2 Inclusion criteria

To participate in this study, a subject must meet all of the following criteria:

1. Adult man;
2. Diagnosed with AUR treated with a transurethral catheter (TUC) and alpha-blocker therapy;
3. Mentally competent and understanding of benefits and potential burden of the study;
4. Written or digital informed consent.

4.3 Exclusion criteria

Patients who meet any of the following criteria will be excluded from participation in this study: a failed prior TWOC, urinary retention >1500ml, usage of an alpha-blocker other than tamsulosin, silodosin or alfuzosin, neurogenic bladder dysfunction (e.g. multiple sclerosis, spinal cord injury, spina bifida, vertebral disc prolapse), history of prostate cancer (ISUP grade group ≥ 2), active bladder cancer or undergoing follow-up for bladder cancer, urinary retention following surgery (within 72 hours post-operation), history of lower urinary tract surgery (e.g., bladder augmentation, urethral surgery, prostate surgery), bladder stones, suspected urethral stricture, clot retention, urosepsis or a contra-indication for alpha blocker therapy.

4.4 Sample size calculation

Based on previous studies (1,8,24), both groups are assumed to have a 50% re-catheterization rate. With a one-sided alpha of 0.05 and a power of 80%, 215 participants per arm are needed to detect a predefined non-inferiority margin. Including an anticipated 10% dropout, the final requirement is 239 participants per arm.

Given an annual incidence of approximately 40,000 AUR cases in the Netherlands and the participation of multiple hospitals, this sample size is considered feasible.

5. TREATMENT OF RESEARCH PARTICIPANTS

5.1 Investigational product/treatment

The proposed intervention involves performing a trial without catheter (TWOC) three days after catheter insertion in men with AUR. This study exclusively includes patients who continue or initiate alpha-blocker therapy as part of standard care. Multiple systematic reviews support the efficacy of alpha-blocker therapy prior to TWOC, leading to its recommendation in several guidelines (20-23). Thus, no medication is administered or tested within the scope of this study.

5.2 Use of co-intervention (if applicable)

Not applicable.

5.3 Escape medication (if applicable)

Not applicable.

6. METHODS

6.1 Study parameters/endpoints

6.1.1 Main study parameter/endpoint

Re-catheterization rate after TWOC.

6.1.2 Secondary study parameters/endpoints (if applicable)

Patient important outcomes:

1. Symptoms measured by 'International Prostate Symptom Score' (IPSS: voiding and storage symptoms) and International Consultation on Incontinence Questionnaire - Long-Term Catheter Quality of Life (ICIQ-LTCQoL: catheter symptoms and concern) on days 3 and 12, and at 1, 2, 3, and 6 months.
2. QoL measured by EuroQol 5 Dimensions, 5 Levels questionnaire (EQ-5D-5L: general health and QoL) on days 3 and 12, and at 1, 2, 3, and 6 months.

Process related outcomes:

Catheter related complications (e.g., macroscopic hematuria, CAUTI, urosepsis, catheter related pain or other catheter related problems, urethral strictures), assessed through medical record review and telephone evaluation. All complications will be classified according to the Clavien-Dindo classification system.

Resource related outcomes:

1. Health care costs (e.g., costs of (re)catheterization, diagnosis and treatment of complications, and hospital admissions) over a 6-month time horizon, assessed through medical record review and the iMTA Medical Consumption Questionnaire (iMCQ) at 3 and 6 months.
2. Social costs: productivity losses over a 1-month time horizon, assessed using the iMTA Productivity Cost Questionnaire (iPCQ) at 1, 2, 3 and 6 months.

6.1.3 Other study parameters

Baseline characteristics will include age, weight, height, medical history (e.g., diabetes mellitus, cardiovascular disease, and neurological disorders), retention volume, and prostate volume (PV).

6.2 Randomization, blinding and treatment allocation

Randomization will be automated and computer-generated using a 1:1 allocation ratio with variable block sizes through a secure, web-based randomization software, ensuring concealed allocation. Blinding of participants and medical staff during the trial is not feasible. To ensure an unbiased assessment, data analysts will remain blinded to the treatment assignments throughout the analysis phase.

6.3 Study procedures

6.3.1 Catheter withdrawal

Detailed recruitment and consent procedures are outlined in Chapter 9.2. Once consent is obtained, either the local or the coordinating investigator will randomize the participant and subsequently notify both the patient and the hospital planning team. All TWOC procedures will be conducted in an outpatient or clinical setting, with participating hospitals scheduling these procedures while adhering to the allocated catheter duration. If a TWOC procedure falls on a weekend, it will be rescheduled to the next working day, with this adjustment applied equally across both groups.

If the TWOC is successful (generally defined as a voided volume over 100 ml and post-void residual volume under 200 ml), re-insertion of the catheter is not required. Patients may either return for follow-up or be discharged, depending on local hospital protocols. In case of an unsuccessful TWOC, a catheter will be reinserted, and the endpoint of re-catheterization is reached. Further steps will generally adhere to the European Association of Urology guidelines (22), although they may vary based on the local team's approach and patient preferences.

6.3.2 Follow-up

The primary outcome measure, re-catheterization following TWOC, will be obtained either 3 or 14 days after initial presentation with AUR. Most secondary outcomes, including patient-reported symptoms, QoL, short-term complications, medical costs, and societal costs, will be evaluated after 1, 2, 3, and 6 months. Long-term complications, along with routine follow-up care, will be monitored through passive data collection over an additional 12 months, yielding a total study duration of 18 months.

Telephone assessment

The investigator will contact participants by phone 30 days after catheter removal / TWOC to document any short-term complications related to catheter use.

Medical record review

Data on long-term complications (primarily strictures) will be collected through medical record review over an 18-month follow-up period. Furthermore, all standard follow-up care participants receive after TWOC will be documented throughout this period.

Questionnaires

Patient-reported symptoms and QoL will be assessed using Dutch versions of three validated questionnaires. The EQ-5D-5L is a general QoL questionnaire evaluating five dimensions of health (25). The IPSS assesses urinary symptoms in patients with BPH (26), whereas the ICIQ-LTCQoL focuses on catheter-related symptoms and concerns (27). Validation of a Dutch version of the latter is currently being conducted. Patients will complete all three questionnaires three days post-catheter insertion for baseline assessment. After 12 days and 1, 2, 3, and 6 months, the IPSS will be administered to patients who no longer have a catheter, while the ICIQ-LTCQoL will be completed by patients with an indwelling catheter.

Two additional questionnaires will be employed to contribute to a cost-effectiveness analysis. The iPCQ quantifies productivity losses resulting from illness (28), and will be completed at 1, 2, 3 and 6 months post-enrollment. The iMCQ will be completed at 3 and 6 months post-enrolment to capture both direct medical costs, such as healthcare and medication use, and non-medical costs, including travel expenses and assistance provided by family or friends (29).

All questionnaires will be completed digitally. For patients who are illiterate or lack digital skills, telephone interviews will be offered as an alternative.

6.4 Withdrawal of individual research participants

Subjects can leave the study at any time for any reason if they wish to do so without any consequences. The investigator can decide to withdraw a subject from the study for urgent

medical reasons, or if the medical condition of the patient changes and in- or exclusion criteria are no longer met.

6.5 Replacement of individual research participants after withdrawal

Subjects withdrawn from the study after recruitment (recruitment defined as signing of the informed consent form) will not be replaced. We anticipated a 10% drop-out rate during our power calculation.

6.6 Follow-up of research participants withdrawn from treatment

Patients withdrawn from the study will be followed-up by their treating physician conform standard treatment procedure.

6.7 Premature termination of the study

Premature termination of the study is not anticipated, as potential re-catheterization following an early catheter removal is not considered a severe medical intervention or complication.

7. SAFETY REPORTING

7.1 Temporary halt for reasons of research participant safety

In accordance to section 10, subsection 4, of the WMO, the sponsor will suspend the study if there is sufficient ground that continuation of the study will jeopardise subject health or safety. The sponsor will notify the accredited METC without undue delay of a temporary halt including the reason for such an action. The study will be suspended pending a further positive decision by the accredited METC. The investigator will take care that all subjects are kept informed.

7.2 AEs, SAEs

7.2.1 Adverse events (AEs)

Adverse events are defined as any undesirable experience occurring to a subject during the study, whether or not considered related to trial procedures. All AEs related to the catheter, TWOC, or potential re-catheterization will be recorded, whether reported spontaneously by the subject or observed by the investigator or his staff.

7.2.2 Serious adverse events (SAEs)

A serious adverse event is any untoward medical occurrence or effect that

- results in death;
- is life threatening (at the time of the event);
- requires hospitalisation or prolongation of existing inpatients' hospitalisation;
- results in persistent or significant disability or incapacity;
- is a congenital anomaly or birth defect; or
- any other important medical event that did not result in any of the outcomes listed above due to medical or surgical intervention but could have been based upon appropriate judgement by the investigator.

An elective hospital admission will not be considered as a serious adverse event.

Due to the high amount of expected (serious) adverse events caused by treatment of the patients as part of standard care, only study related (S)EAs will be reported immediately. This means all (S)AEs related to participation in this study protocol,

meaning an early TWOC procedure and potential re-catheterization, will be reported. All other (S)AEs will not be reported, as no patient benefit is expected from this.

Local investigators report all study-related SAEs to the sponsor without undue delay after obtaining knowledge of the events. The sponsor will subsequently report the SAEs through the web portal *Research Portal* to the accredited METC that approved the protocol, within 7 days of first knowledge for SAEs that result in death or are life threatening followed by a period of maximum of 8 days to complete the initial preliminary report. All other SAEs will be reported within a period of maximum 15 days after the sponsor has first knowledge of the serious adverse events.

7.3 Follow-up of adverse events

All AEs will be followed until they have abated, or until a stable situation has been reached. Depending on the event, follow up may require additional tests or medical procedures as indicated, and/or referral to the general physician or a medical specialist.
SAEs need to be reported till end of study within the Netherlands, as defined in the protocol.

7.4 Data Safety Monitoring Board (DSMB) / Safety Committee

Not applicable.

8. STATISTICAL ANALYSIS

Statistical analyses will be performed using SPSS (IBM, version 26). All analyses will be two-sided, and a p-value of less than 0.05 will be considered indicative of statistical significance. Baseline characteristics will be summarized and reported using frequency (percentage) for categorical variables, and mean ± standard deviation (SD) or median (interquartile range, IQR) for continuous variables.
Randomization minimizes confounding. If any imbalance arises in baseline covariates, adjusted analyses will be performed.
Due to potential loss to follow-up, we anticipate some missing data. We will assume the data is missing at random and handle it using multiple imputation with chained equations. This method will create 25 imputed datasets, with results subsequently pooled using Rubin's rules to ensure reliable analysis.

8.1 Primary study parameter(s)

For the primary outcome of this RCT, we will compare the percentage of patients undergoing re-catheterization after unsuccessful TWOC. Primarily, an intention-to-treat analysis will be performed, followed by an additional per-protocol analysis. If the 95% confidence interval for the difference in re-catheterization rates lies entirely within 12% (non-inferiority margin, as described in chapter 4.4), non-inferiority of the 3-day duration is declared. A mixed-effects model will be employed to account for the clustered data from the different participating hospitals.

8.2 Secondary study parameter(s)

<u>Complications</u>
Complication rates at 21 days, 6 months, 12 and 18 months (e.g., hematuria, infection) will be compared using Fisher’s exact test. This statistical test will help determine if there is a significant disparity in complication rates.

<u>Patient reported outcomes</u>
Changes in QoL (measured by IPSS-Qol, ICIQ-LTCQoL, and EQ-5D-5L) will be analyzed with longitudinal covariance analysis. This analytical approach will enable us to investigate the evolution of QoL over time in the different treatment groups.

Cost analysis

The economic evaluation will be performed alongside the clinical trial and is designed as a cost-effectiveness analysis from a societal perspective. The analysis will take a time horizon of 6 months after enrolment in the study. Bootstrapping will be used to calculate the distribution of the ICER, which will be shown in a cost-utility plane.

8.3 Other study parameters

Not applicable.

8.4 Interim analysis (if applicable)

No interim analysis is planned to minimize type I error (incorrectly rejecting the null hypothesis when it is true).

9. ETHICAL CONSIDERATIONS

9.1 Regulation statement

The study will be conducted according to the principles of the Declaration of Helsinki (date 13th of December 2024, see for the most recent version: www.wma.net) and in accordance with the Medical Research Involving Human Subjects Act (WMO) and other guidelines, regulations and Acts.

9.2 Recruitment and consent

9.2.1 Recruitment

Participants will be identified upon presentation with AUR, either in the emergency department or at the urological outpatient clinic. Alternatively, patients may be enrolled following referral from their general practitioner or the GP emergency service (in Dutch: Huisartsenpost).

9.2.2 Informed consent procedures

After catheterization, eligible patients will be informed about the study and receive a written Patient Information Form (PIF) from the attending physician. With the patient's permission, contact details will be shared with the coordinating investigator or a delegated member of the study team, who will subsequently contact the patient by phone to answer any questions and provide further clarification. Detailed information about the study objectives and procedures is additionally available on the study's website, along with an informative video. Each subject must be informed that participation in the study is voluntary and that refusal to provide consent will not affect the doctor-patient relationship.
Given the acute nature of the condition and the potential need for a TWOC within three days, an efficient informed consent process is required. The traditional method of signing the ICF at home and returning it by postal mail is not feasible, as this process would exceed the three-day timeframe.

Once the patient has received the study information, they will be given at least 24 hours to consider participation. Following this reflection period, informed consent may be provided either by physically submitting the signed Informed Consent Form (ICF) to the hospital, or by completing a digital informed consent procedure via the

secure REDCap system. In the case of written consent, the local principal investigator or a delegated member of the study team will co-sign the ICF upon receipt. For digital consent, the coordinating investigator or a delegated team member will electronically countersign the form within the REDCap environment. Randomization will only occur after both the patient and the local or coordinating investigator (or their delegate) have signed the informed consent form.

9.2.3 Digital informed consent

In compliance with privacy regulations, REDCap -a secure web-based application for managing online databases- is utilized. REDCap includes a specialized module that enables the secure transmission of digital PIFs and ICFs. Participants will provide their digital signatures using a computer mouse or touchscreen, depending on their device.

To ensure the validity of electronic signatures, three key conditions are met:

1. Identification. Digital ICFs will be provided exclusively to eligible patients following identification by their attending physician. Patients' details, including email, will be securely transmitted to the coordinating investigator to ensure the correct individual receives the form. Access to the ICF will be restricted through two-factor authentication, utilizing the provided, personal email address.
2. Document integrity. Signed forms are stored as immutable PDFs, preventing alterations. Participants receive electronic copies for their records.
3. Timestamping. As REDCap includes an audit trail feature, all data related to the signing process will be systematically recorded.

Study-related data will not be accessible to the service provider. Each participant will be assigned a unique REDCaP study ID. The Subject Identification Code List, which contains the participant's name, date of birth, and email address, will be stored separately from other study data. Access to this list will be restricted to study team members involved in study management.

Details regarding the reliability and confidentiality of the e-consent process are provided in document 'K6. E-consent Questions and Answers CCMO-UMCG'.

9.3 Benefits and risks assessment, group relatedness

The primary treatment for AUR is to drain the bladder using a TUC. This procedure offers immediate pain relief, decompresses the bladder, and prevents potential renal damage. It is regarded as a safe procedure and can be performed by trained nurses, GPs, (emergency) doctors, or urologists. TWOC is defined as removal of the catheter with subsequent observation of spontaneous micturition.

Both study groups will undergo a TWOC, differing solely in timing. A 14-day catheterization duration aligns closely with the median duration observed in Dutch clinical practice (11). A potential burden for participants in the study group may be a higher likelihood of unsuccessful TWOC, leading to more frequent re-catheterization. However, the health risks associated with re-catheterization are considered to be of low impact or rare. Moreover, multiple studies have indicated that prolonged catheterization is associated with an increased risk of complications (6,7,8,10,12,13). Thus, shorter catheterization may reduce discomfort and complications without adversely affecting TWOC success.

9.4 Compensation for injury

The sponsor/investigator has a liability insurance which is in accordance with article 7 of the WMO. The sponsor (also) has an insurance which is in accordance with the legal requirements in the Netherlands (Article 7 WMO). This insurance provides cover for damage to research participants through injury or death caused by the study. The insurance applies to the damage that becomes apparent during the study or within 4 years after the end of the study.

9.5 Incentives (if applicable)

Not applicable.

10. ADMINISTRATIVE ASPECTS, MONITORING AND PUBLICATION

10.1 Handling and storage of data and documents

The handling of personal data complies with EU General Data Protection Regulation (GDPR) and the Dutch Act on Implementation of the General Data Protection Regulation (in Dutch: Uitvoeringswet AVG). A data management plan has been established, outlining procedures and protocols to ensure data integrity and compliance with EU GDPR.
All collected data will be anonymized; however, patient identities will be accessible via a code list, as this is necessary for data collection. This list will be securely stored securely on the hospital’s internal network, accessible only to the coordinating investigator and local researchers. Patient coding will not involve the use of initials or dates of birth, and the code key will be securely kept by the investigator.
Physical ICFs will be securely stored in a locked cabinet at the Department of Urology, accessible only to authorized personnel, while digital ICFs will be stored in the ReDCap system. Patients will receive a copy of the ICF via mail or email.
Data will be retained for 15 years after study follow-up ends, and data collection will be managed through Research Manager.

10.2 Monitoring and Quality Assurance

Monitoring will be conducted in accordance with the NFU (Nederlandse Federatie van Universitair Medische Centra) guideline "Quality Assurance of Human Research 2.0". Independent, qualified monitors employed by the department of Innovation and Science at Isala will oversee the monitoring of the study. The risk classification for this study is considered "negligible", requiring thorough monitoring with a minimum of one visit each year. Additional visits may be conducted depending on the inclusion rate and any previously observed deviations.

The monitor will verify compliance with standard operating procedures (SOPs), ensure the presence of mandatory documents, and perform source data verification on the research data. Any findings identified during the monitoring visits will be communicated to the sponsor-investigator through a detailed monitoring visit report. The sponsor-investigator is responsible for addressing any findings, deviations, inquiries, or issues that arise and ensuring that appropriate follow-up actions are implemented.

10.3 Amendments

Amendments are changes made to the research after a favourable opinion by the review committee has been given. All amendments will be notified to the review committee that gave a favourable opinion. Non-substantial amendments will not be notified to the review committee, but will be recorded and filed by the sponsor.

10.4 Annual progress report

The sponsor/investigator will submit a summary of the progress of the trial to the review committee once a year. Information will be provided on the date of inclusion of the first participant, numbers of participants included and numbers of participants that have completed the trial, serious adverse events, other problems, and amendments.

10.5 Temporary halt and (prematurely) end of study report

The investigator/sponsor will notify the review committee of the end of the study within a period of 8 weeks. The end of the study is defined as the end of the 18 month follow-up period of the last enrolled patient.
The sponsor will notify the review committee immediately of a temporary halt of the study, including the reason of such an action.
In case the study is ended prematurely, the sponsor will notify the review committee within 15 days, including the reasons for the premature termination.
Within one year after the end of the study, the investigator/sponsor will submit a final study report with the results of the study, including any publications/abstracts of the study, to the review committee.

10.6 Public disclosure and publication policy

Data publication will adhere to CCMO guidelines, with all findings shared, regardless of the hypothesis outcome. Individual results will remain confidential. Efforts will be made to publish in peer-reviewed journals and present results at meetings, symposia and conferences.

11. REFERENCES

1. D'Ancona CD, et al. An International Continence Society (ICS) report on the terminology for adult male lower urinary tract and pelvic floor symptoms and dysfunction. Neurourol Urodyn. 2019.
2. Nederlandse Vereniging voor Urologie. Kennisagenda van de NVU 2020-2024. Available from: https://demedischspecialist.nl/sites/default/files/Kennisagenda%20Urologie%202020-2024.pdf
3. Yoon PD, Chalasani V, Woo HH. Systematic review and meta-analysis on management of acute urinary retention. Prostate Cancer Prostatic Dis. 2015 Dec;18(4):297-302. doi: 10.1038/pcan.2015.15.
4. Taube M, Gajraj H. Trial without catheter following acute retention of urine. Br J Urol. 1989 Feb;63(2):180-2. doi: 10.1111/j.1464-410x.1989.tb05160.x
5. Djavan B, Shariat S, Omar M, Roehrborn C, Marberger M. Does prolonged catheter drainage improve the chance of recovering voluntary voiding after acute urinary retention (AUR)? Eur Urol. 1998;33:110.
6. Desgrandchamps F, De La Taille A, Doublet JD; RetenFrance Study Group. The management of acute urinary retention in France: a cross-sectional survey in 2618 men with benign prostatic hyperplasia. BJU Int. 2006 Apr;97(4):727-33. doi: 10.1111/j.1464-410X.2006.06109.x.
7. Christensen VS, Skow M, Flottorp SA, Strømme H, Mdala I, Vallersnes OM. Immediate or delayed trial without catheter in acute urinary retention in males: A systematic review. BJUI Compass. 2024;5(8).
8. Fitzpatrick JM, Desgrandchamps F, Adjali K, Gomez Guerra L, Hong SJ, El Khalid S, Ratana-Olarn K; Reten-World Study Group. Management of acute urinary retention: a worldwide survey of 6074 men with benign prostatic hyperplasia. BJU Int. 2012 Jan;109(1):88-95.
9. Park K, Kim SH, Ahn SG, Lee SJ, Ha US, Koh JS, Lee YS, Han CH, Cho SY, Kim HW. Analysis of the treatment of two types of acute urinary retention. Korean J Urol. 2012 Dec;53(12):843-7. doi: 10.4111/kju.2012.53.12.843.
10. Abuelnaga M, Elawady H, Mahmoud MA, Mostafa D, Samir M. Does the duration of catheterization have an impact on the outcome of trial without catheter in patients with acute urine retention (AUR) due to benign prostatic hyperplasia (BPH)? A prospective randomized study. Urologia. 2023 Dec 4:3915603231215936

11. van Merode NAM, et al. De praktijkvariatie van katheterduur bij mannen met een acute urineretentie: een update van de AUR-SNAPSHOT studie. Tijdschr Urol. 2023; Supplement Voorjaarsvergadering, bijlage 1.
12. Li F, Song M, Xu L, Deng B, Zhu S, Li X. Risk factors for catheter-associated urinary tract infection among hospitalized patients: A systematic review and meta-analysis of observational studies. J Adv Nurs. 2019 Mar;75(3):517-527. doi: 10.1111/jan.13863.
13. Letica-Kriegel AS, Salmasian H, Vawdrey DK, Youngerman BE, Green RA, Furuya EY, Calfee DP, Perotte R. Identifying the risk factors for catheter-associated urinary tract infections: a large cross-sectional study of six hospitals. BMJ Open. 2019 Feb 21;9(2):e022137. doi: 10.1136/bmjopen-2018-022137.
14. Fisher E, Subramonian K, Omar MI. The role of alpha blockers prior to removal of urethral catheter for acute urinary retention in men. Cochrane Database Syst Rev. 2014 Jun 10;(6):CD006744. doi: 10.1002/14651858.CD006744.pub3.
15. Gwon YN, et al. Comparing effects of alpha-blocker management on acute urinary retention secondary to benign prostatic hyperplasia: A systematic review and network meta-analysis. Prostate Int. 2023 Jun;11(2):91-99.
16. Geneesmiddeleninformatiebank. Summary of Product Characteristics (SmPC) tamsulosin. Cited 2025 Apr 4. Available from: https://www.geneesmiddeleninformatiebank.nl/smpc/h106165_smpc.pdf
17. Geneesmiddeleninformatiebank. Summary of Product Characteristics (SmPC) silodosin. Cited 2025 Apr 4. Available from: https://www.geneesmiddeleninformatiebank.nl/smpc/h123018_smpc.pdf
18. Geneesmiddeleninformatiebank. . Summary of Product Characteristics (SmPC) alfuzosin. Cited 2025 Apr 4. Available from: https://www.geneesmiddeleninformatiebank.nl/smpc/h31128_smpc.pdf
19. Karavitakis M, et al. Management of urinary retention in patients with benign prostatic obstruction: A systematic review and meta-analysis. Eur Urol. 2019;75:788.
20. Lerner LB, McVary KT, Barry MJ, Bixler BR, Dahm P, Das AK, Gandhi MC, Kaplan SA, Kohler TS, Martin L, Parsons JK, Roehrborn CG, Stoffel JT, Welliver C, Wilt TJ. Management of lower urinary tract symptoms attributed to benign prostatic hyperplasia: AUA guideline part I—initial work-up and medical management. J Urol. 2021 Oct;206(4):806-817.
21. National Institute for Health and Care Excellence (NICE). Lower urinary tract symptoms in men: management. NICE Clinical Guideline CG97. Published May 2010; last updated June 2015. Available from: https://www.nice.org.uk/guidance/cg97

22. Gravas S, et al. EAU guidelines on the assessment of non-neurogenic male lower urinary tract symptoms including benign prostatic obstruction. Eur Urol. 2015;68(3):438-50. doi: 10.1016/j.eururo.2015.01.024.
23. Blanker MH, Breed SA, van der Heide W, Norg RJC, de Vries A, Wolters RJ, van den Donk M, Burgers JS, Opstelten W, Klomp MA. NHG-Standaard Mictieklachten bij mannen. Huisarts Wet. 2013;56(3):114-22. Available from: https://richtlijnen.nhg.org/standaarden/mictieNVU
24. Nederlandse Vereniging voor Urologie (NVU). Richtlijn ‘’Mannelijke niet-neurogene LUTS’’ (Guideline for Male Non-Neurogenic Lower Urinary Tract Symptoms). Published 2012; last updated Nov 2017 [cited 2025 Apr 4]. Available from: https://richtlijnendatabase.nl/richtlijn/mannelijke_niet-neurogene_luts/startpagina_luts.html
25. Herdman M, Gudex C, Lloyd A, Janssen MF, Kind P, Parkin D, Bonsel G, Badia X. Development and preliminary testing of the new five-level version of EQ-5D (EQ-5D-5L). Qual Life Res. 2011;20(10):1727-1736. doi: 10.1007/s11136-011-9903-x.
26. Badía X, García-Losa M, Dal-Ré R. Ten-language translation and harmonization of the International Prostate Symptom Score: developing a methodology for multinational clinical trials. Eur Urol. 1997;31(2):129-40.
27. Cotterill N, Fowler S, Avery M, Cottenden A, Wilde M, Long A, Fader M. Development and psychometric evaluation of the ICIQ-LTCqol: a self-report quality of life questionnaire for long-term indwelling catheter users. Neurourol Urodyn. 2016;35(3):423-428.
28. Bouwmans C, Krol M, Severens H, Koopmanschap M, Brouwer W, Hakkaart-van Roijen L. (2015) The IMTA Productivity Cost Questionnaire: A standardized instrument for measuring and valuing health-related productivity losses. Value in Health. 18:753-758
29. iMTA Productivity and Health Research Group. Manual iMTA Medical Cost Questionnaire (iMCQ). Rotterdam: iMTA, Erasmus University Rotterdam, 2018. Available from: https://www.imta.nl/questionnaires/imcq/publications/.
